# Steven-Johnson Syndrome Induced by Lamotrigine and Valproic Acid in a Pediatric Patient: A Case Report

**DOI:** 10.7759/cureus.41267

**Published:** 2023-07-01

**Authors:** Eunice-Jazmín Espinosa-Aguilar, Steven-Andrés Piña-Ballantyne, Keren-Lizeth Espinosa-Aguilar, Juan-Carlos Tun-Pisté, Ana-Laura Calderón-Garcidueñas

**Affiliations:** 1 Internal Medicine, Clínica-Hospital Mérida, Mérida, MEX; 2 Neuropathology, Instituto Nacional de Neurología y Neurocirugía Manuel Velasco Suárez, Mexico City, MEX; 3 Pediatrics, Hospital General de Especialidades "Dr. Javier Buenfil Osorio", Campeche, MEX; 4 Internal Medicine, Hospital General de Especialidades "Dr. Javier Buenfil Osorio", Campeche, MEX

**Keywords:** antiseizure medications, steven-johnson syndrome (sjs), toxic epidermal necrolysis (ten), valproic acid, lamotrigine

## Abstract

Steven-Johnson syndrome (SJS) and toxic epidermal necrolysis (TEN) are part of a spectrum of severe cutaneous adverse reactions, secondary to infections or drug-induced. Although the use of antiseizure medications (ASMs) is a risk factor for the development of SJS/TEN, primary care physicians are not familiar with these cases in some countries. We report a case of SJS associated with ASMs in a nine-year-old girl with a history of difficult-to-control epilepsy, who required adjustment and change in medications.

## Introduction

Steven-Johnson syndrome (SJS) and toxic epidermal necrolysis (TEN) are rare dermatological emergencies characterized by epidermal necrolysis and epidermal and mucous membrane detachment; the difference between these pathologies is the extension of total body surface area (BSA) involved, since in SJS, it is <10% of total BSA and in TEN, it is > 30% [1]. They are associated with the perforin/granzyme B-induced (P/GrB) apoptotic pathway used by cytotoxic lymphocytes (CD8+) to eliminate virus-infected cells [2]. The onset of SJS/TEN may be induced as an adverse reaction to drugs, associated with infections, or may be idiopathic. Among the adverse reactions to medications, the use of antiseizure medication (ASM) stands out.

Most cases of SJS and TEN induced by ASM have been reported after puberty and in young adults, with rare cases of occurrences in infancy or early childhood [3]. Early diagnosis by first-contact doctors is essential for proper management. We present a case of a nine-year-old girl with SJS associated with ASM.

## Case presentation

The patient, a nine-year-old Mexican girl with dyslexia, had a chikungunya infection at eight months of age and presented with tonic-clonic febrile epileptic seizures. Subsequently, the patient continued to experience seizures, which required specialized attention from a pediatric neurologist. Electroencephalogram showed focal temporal lobe epilepsy and mild cognitive disability. Cranial computed tomography (CT) without contrast did not show any brain structural lesions. The patient was initially treated with valproic acid (VPA) (300-200-300 mg/8 hours) and four months later, carbamazepine (CBZ) (200 mg/8 hours) was added. Despite dual antiseizure treatment for the past eight years, she had multiple hospitalizations due to a lack of epilepsy control, presenting at least one seizure episode every two months.

**Figure 1:**
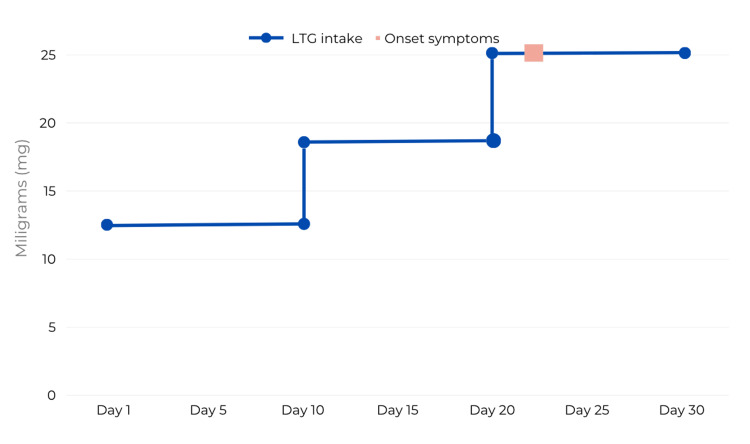
Temporal time frame of LTG administration LTG: Lamotrigine.

At the age of nine years, CBZ was replaced with lamotrigine (LTG). The initial dose was a half-tablet of a 25 mg dispersible tablet every 12 hours during the first two weeks. This dose was increased after 10 days, to three-fourths of a tablet every 12 hours, and 10 days later, the entire tablet every 12 hours (Figure 1).

Symptoms started three weeks after the initial LTG dose, with conjunctival hyperemia, bilateral purulent discharge, and general malaise. Her parents administered chamomile eye drops without any improvement. It progressed to localized dermatosis and edema on her face and mouth, lip hyperemia, and oral mucosal ulcers. She was treated ambulatory with loratadine, neomycin eye drops, and polymyxin B topical ointment by her family doctor. Subsequently, genital dermatosis with painful blisters appeared, and oral mucosal ulcers increased, causing intense pain and oral intolerance. Non-painful and non-pruritic erythematous macules were observed in the anterior and posterior thorax and the upper extremities. Due to worsening symptoms, the patient was transferred to the emergency room.

**Figure 2:**
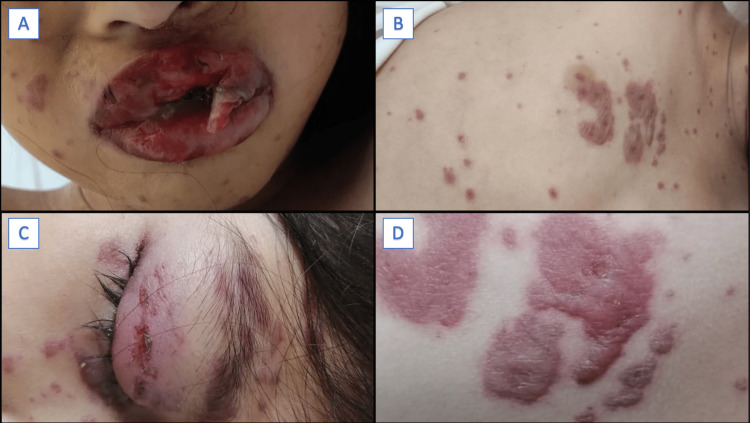
Dermatological examination at initial presentation Edema, erythema, ulcerated, and painful lesions around and inside the oral mucosa (A) and on the eyelids (C). Thorax  showing erythematous-violaceous polymorphic plaques of different sizes (B, D).

On admission, her face showed palpebral edema and erythema with abrasions covered by bloody crusts. The perioral area was swollen, with red areas of abrasion, edema, and erythema of the lips, and numerous superficial ulcers were observed in the oral mucosa. The anterior and posterior thorax showed erythematous, violaceous, and polymorphic plaques of different sizes with a tendency to merge (Figure 2). The external genitalia had ulcerative lesions in the labia majora, minora, vulva, perineum, and perianal area.

**Figure 3:**
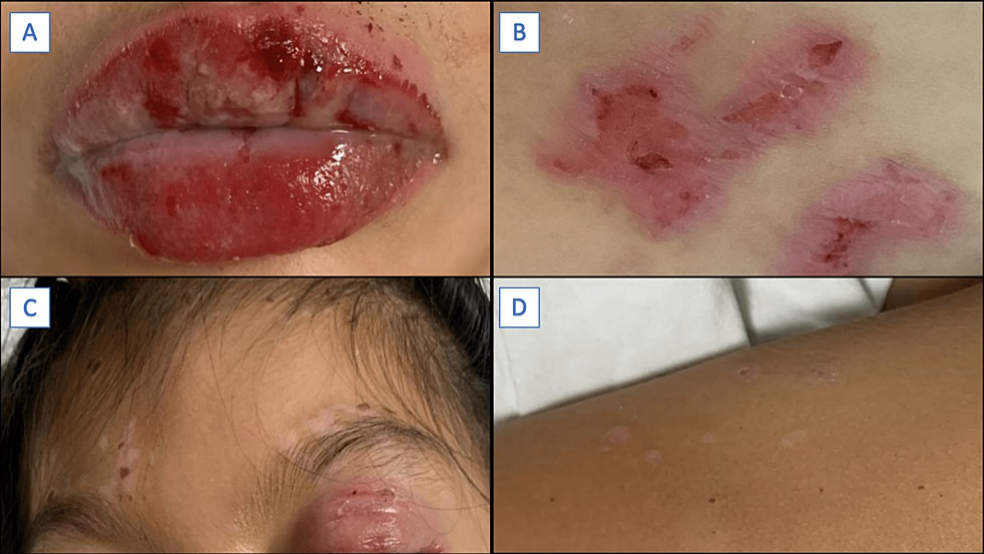
Dermatological examination after five days of treatment Clinical improvement of oral (A) and palpebral lesions (C), as well as involution of thoracic lesions (B), with some residual hypopigmented spots (D) were observed.

The affected mucocutaneous surface was <10%; therefore, SJS was diagnosed. Initial basic laboratory examination (complete blood count, blood chemistry, liver profile, and serum electrolytes) results were normal. Fluid management and intravenous (IV) administration of methylprednisolone (1 mg/kg/day for five days) and human immunoglobulin G (IgG) (2 g/kg/12 hours for three days) were initiated. During the first 24 hours of admission, fever of 38°C was documented, and third-generation cephalosporin (ceftriaxone 100 mg/kg/day) was administered. Philadelphia Mouthwash topical solution was used to treat oral lesions, and saline water and soap washes for skin lesions. She was evaluated ophthalmologically and bilateral keratitis was diagnosed, which required management with hypromellose and tobramycin eye drops. During hospitalization, her symptoms and skin lesions improved (Figure 3).

The patient was discharged on the ninth day of hospitalization with topical treatment. Given that she had been treated with VPA for eight years without presenting with adverse reactions, it was decided to discontinue LTG upon admission and continued only with VPA until her appointment in pediatric neurology. No epileptic seizures were documented during her hospitalization.

## Discussion

SJS was first defined by Albert Stevens and Frank Johnson in 1922 [4]. The syndrome has a worldwide distribution, affecting all races, with a women: men ratio of 2:1 [4, 5]. Its incidence is 2-13 per million patients per year [6]. Mortality rate is 4.8-9%, with sepsis being the main cause [1, 7]. Regarding the pediatric population, Hsu et al. reported in the United States, an incidence rate of 5.3 and 0.4 cases, per million children for SJS and TEN, respectively [3].

SJS is an autoimmune type IV hypersensitivity reaction characterized by epidermal and mucous membrane detachment due to extensive keratinocyte death that affects <10% of the skin surface [6, 8]. According to Wallace’s rule of nine, the patient's palm represents 1% of the affected skin [9]. Patients with severe drug reactions are exposed to increased amounts of oxidative metabolites secondary to a decreased ability to detoxify reactive metabolites, either for genetic or functional reasons [2].

SJS is associated with infection and adverse drug effects. Infectious triggers include herpes simplex and Mycoplasma pneumoniae infections. Drugs include sulfonamides, antibiotics, nonsteroidal anti-inflammatory drugs (NSAIDs) and ASM. Idiopathic cases have also been reported [5, 7]. In some cases, ASMs are used in combination, leading to more potential pharmacokinetic and pharmacodynamic interactions [9].

LTG is a phenyltriazine derivative administered orally with complete absorption in the gastrointestinal tract, plasma half-life of a single dose of approximately 24 hours, hepatic metabolism by glucuronidation, and <10% excretion of the parent drug, through the kidneys [10, 11]. LGT and VPA are metabolized in the liver by glucuronosyl-transferase (isoenzyme, UGT1A4). UGT enzyme (UGT1A4) has a higher affinity for VPA; therefore, the metabolism of LTG (also by glucuronidation) is reduced (competition) and the plasma levels increase [12].

Severe cutaneous adverse reactions (SCARs) include SJS and TEN. These pathologies are not dose-dependent but are hypersensitivity syndromes. These have been linked to different human leukocyte antigen (HLA) subtypes, which vary among different ethnic groups. Therefore, SCARs are not predictable. In contrast, adverse drug reactions (ADRs) depend on the drug itself and dosage [13, 14]. Lower initial and maintenance doses of LTG are recommended when prescribed along with other ASMs to minimize the risk of ADRs due to high plasma drug levels [9]. 

In clinical series, it has been shown that the combination of these two drugs can increase the risk of SJS [15]. An analysis of SJS/TEN spectrum case reports by Wang et al. identified 957 medication-related cases (90.3%). Of these, 196 (18.5%) were associated with ASMs and LTG was identified in 49 patients [16]. According to the largest review of drug-associated SJS/TEN in pediatric patients based on a worldwide spontaneous reporting system by Egunsola et al., 486 cases (0-17 years) received LGT and experienced SJS/TEN. Of all SJS/TEN cases, 207 were reported in combination with VPA (43%) and 158 were administered alone (33%) [9]. A systematic review of the safety of LTG in pediatric patients showed that rash was the most common ADRs, occurring in 7.3% of the cases. In almost half (48%) of rash cases, LTG was co-administered with VPA [17].

In our case, the symptoms began three weeks after LTG intake, matching the expected time when these reactions generally appeared (first eight weeks). The lesions did not progress, and symptoms improved after the suspension of LTG. Diagnosis is clinical, using three elements as a guide: skin manifestations, mucosal changes, and histopathological findings from skin biopsy [1]. Histologically, these features include full-thickness epidermal necrolysis due to extensive keratinocyte apoptosis [3]. In our case, the parents refused the biopsy.

The clinical manifestations of SJS are characterized by a prodromal stage, with fever, malaise, sore throat, and cough. The subsequent involvement of the skin and mucosa typically appears as erythematous macules or atypical target lesions on the trunk that progress to confluent areas of erythema with dark centers, flaccid bullae with a positive Nikolsky sign, and sheets of denuded epidermis, affecting <10% of the total body surface. Oral involvement is common, with mucositis and ulceration occurring in up to 100% of the cases [1]. In the acute phase the ocular involvement is between 50-88% of the cases [18], incluiding acute conjunctivitis, eyelid edema, erythema, crusts, and ocular discharge, to conjunctival membrane or pseduomembrane formation or corneal erosion, and, in severe cases, to cicatrizing lesions, symblepharon, fornix foreshortening, and corneal ulceration [19]. Chronic ocular sequeales occur in up to 35% of the cases [18]. 

According to a recent meta-analysis study, in primary care setting, the pooled prevalence of ADRs was 8.32% [20]. Adverse reactions are common, although mild and transient reactions prevail. The treatment of SJS/TEN includes discontinuation of the drug associated with the problem and supportive care. However, the use of systemic corticosteroids, IV immunoglobulin, cyclosporine, and TNF-α antagonists remains controversial [1].

## Conclusions

In conclusion, hypersensitivity reactions are a risk when ASMs are administered; therefore, close monitoring is required during the first two months, since it is the period with the highest number of adverse reactions. Family members should be informed of how to recognize possible adverse effects. SJS is a potentially life-threatening condition that requires immediate intervention and multidisciplinary approach. Therefore, a team of well-trained health personnel is required to prevent and detect possible complications.
